# Supplementary material for: Syndiotactic Poly(4-methyl-1-pentene)-Based Stereoregular Diblock Copolymers: Synthesis and Self-Assembly Studies
Source: Polymers (Basel). 2022 Nov 9;14(22):4815. doi: 10.3390/polym14224815 (PMC9694553; doi:10.3390/polym14224815)
Supplement: Supplementary file 1 [file polymers-14-04815-s001.zip › polymers-2012643-supplementary.pdf]

Syndiotactic Poly(4-methyl-1-pentene)-based Stereoregular Diblock Copolymers: Synthesis and Self-assembly Studies

Yu-Chuan Sung [1], Pei-Sun Huang [1], Shih-Hung Huang [2], Yeo-Wan Chiang [2,*] and Jing-Cherng Tsai [1,*]

[1] Department of Chemical Engineering, National Chung Cheng University
Chiayi 62142, Taiwan

[2] Department of Materials and Optoelectronic Science, National Sun Yat-Sen University, Kaohsiung 80424, Taiwan

* Correspondence to: chmjct@ccu.edu.tw

Supporting information

Figure S1. ^{13}C-NMR and DEPT135 spectra of OH-capped sP4M1P…………....3

Figure S2. ^{1}H-^{13}C HMQC spectrum of OH-capped sP4M1P……………………4

Figure S3. ^{13}C-NMR and DEPT135 spectra of sP4M1P-bromoester……………5

Figure S4. ^{1}H-^{13}C HMQC spectrum of sP4M1P-bromoester……………………6

Figure S5. ^{13}C-NMR and DEPT135 spectra of sP4M1P-*b*-PMMA……………...7

Figure S6. ^{1}H-^{13}C HMQC spectrum of sP4M1P-*b*-PMMA……………………..8

Figure S7. ^{13}C-NMR and DEPT135 spectra of sP4M1P-*b*-aPS………………...9

Figure S8. ^{1}H-^{13}C HMQC spectrum of sP4M1P-*b*-aPS………………………..10

Figure S9. DSC heating thermograms of sP4M1P-*b*-PMMA…………………..11

Figure S10. DSC heating thermograms of sP4M1P-*b*-aPS……………………..12

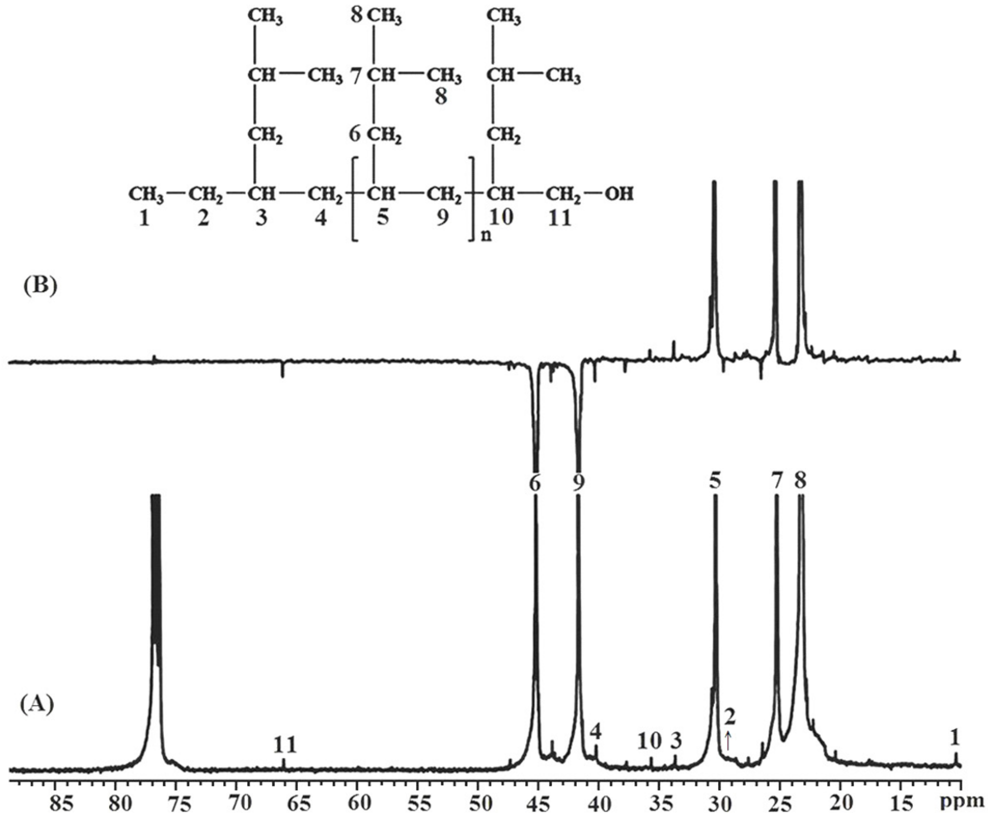

Figure S1. ^{13}C and ^{13}C (DEPT135) NMR spectra (125 MHz) of OH-capped sP4M1P (M_n =9890 g/mol, M_w/M_n=1.65) (solvent: $CDCl_3$; temperature: 60 °C).

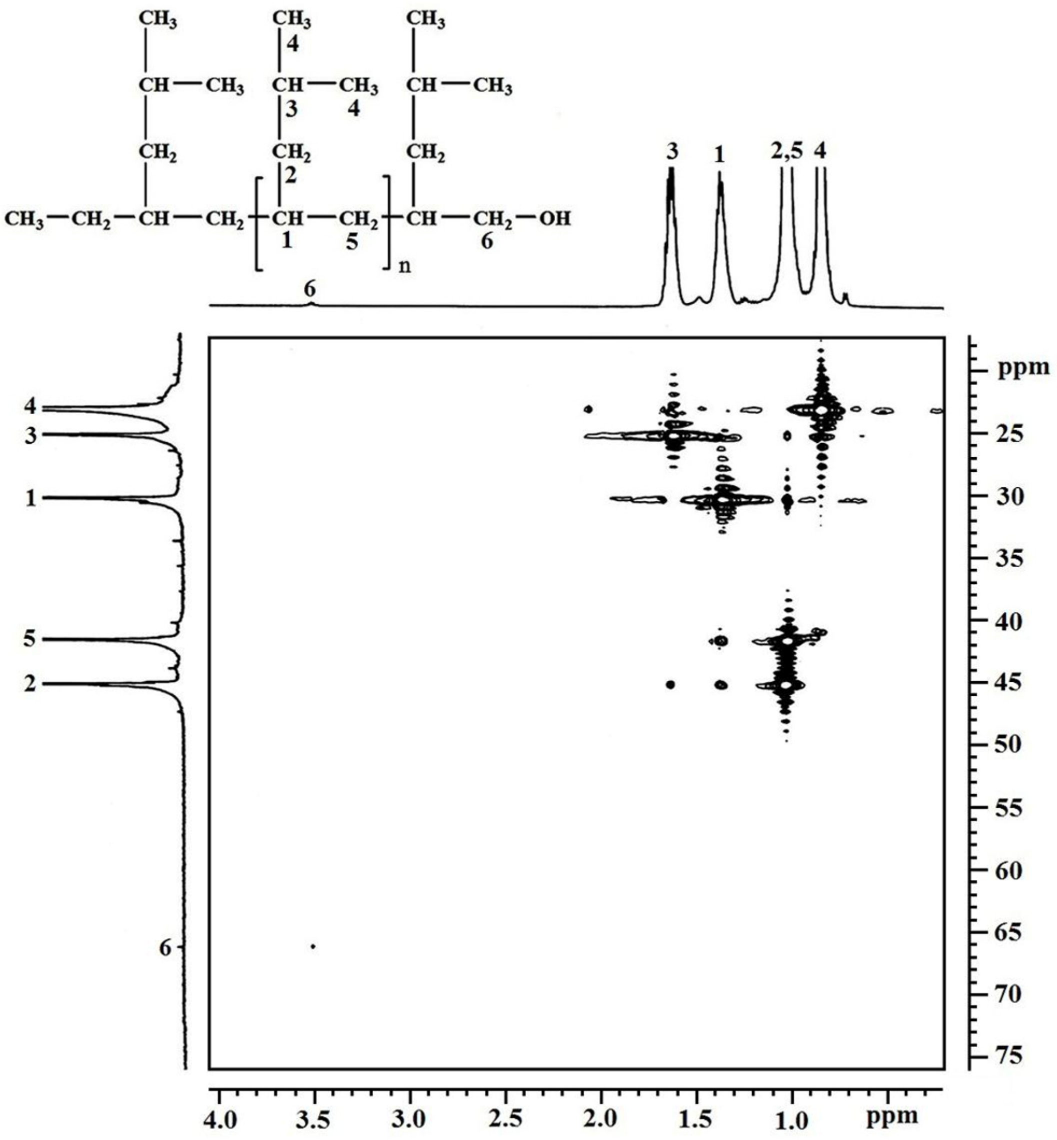

Figure S2. ^{1}H-^{13}C HMQC spectrum of OH-capped sP4M1P (M_n =9890 g/mol, M_w/M_n=1.65) (solvent: $CDCl_3$; temperature: 60°C).

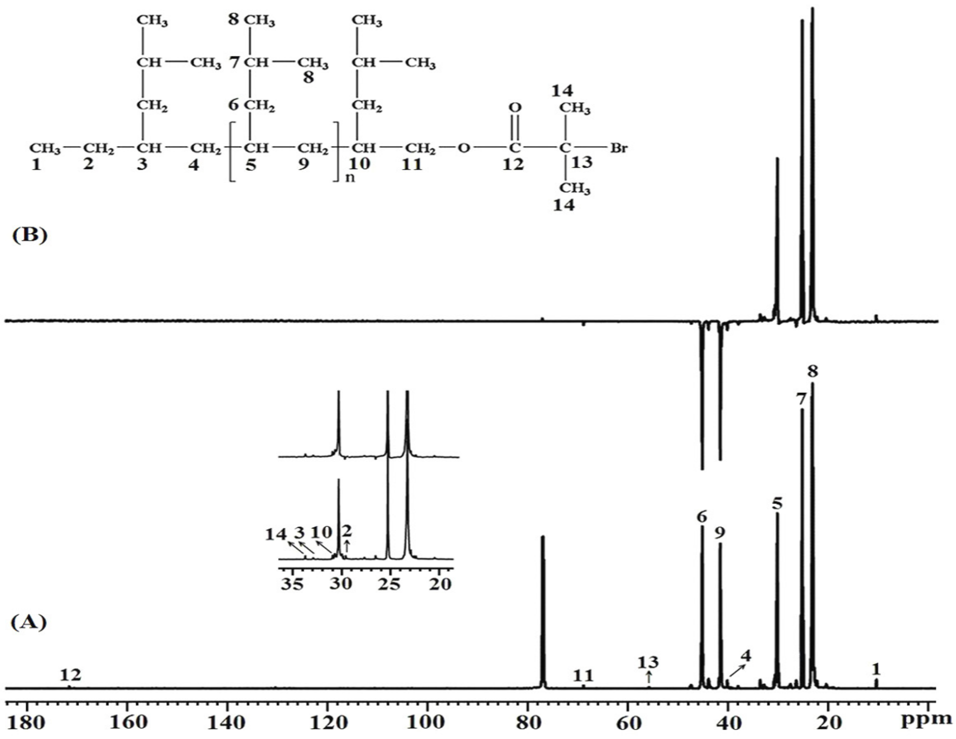

Figure S3. ${}^{13}C$ and ${}^{13}C$ (DEPT135) NMR spectra (125 MHz) of sP4M1P-bromoester (M_n = 10500 g/mol, M_w/M_n = 1.39) (solvent: $CDCl_3$; temperature: 60°C).

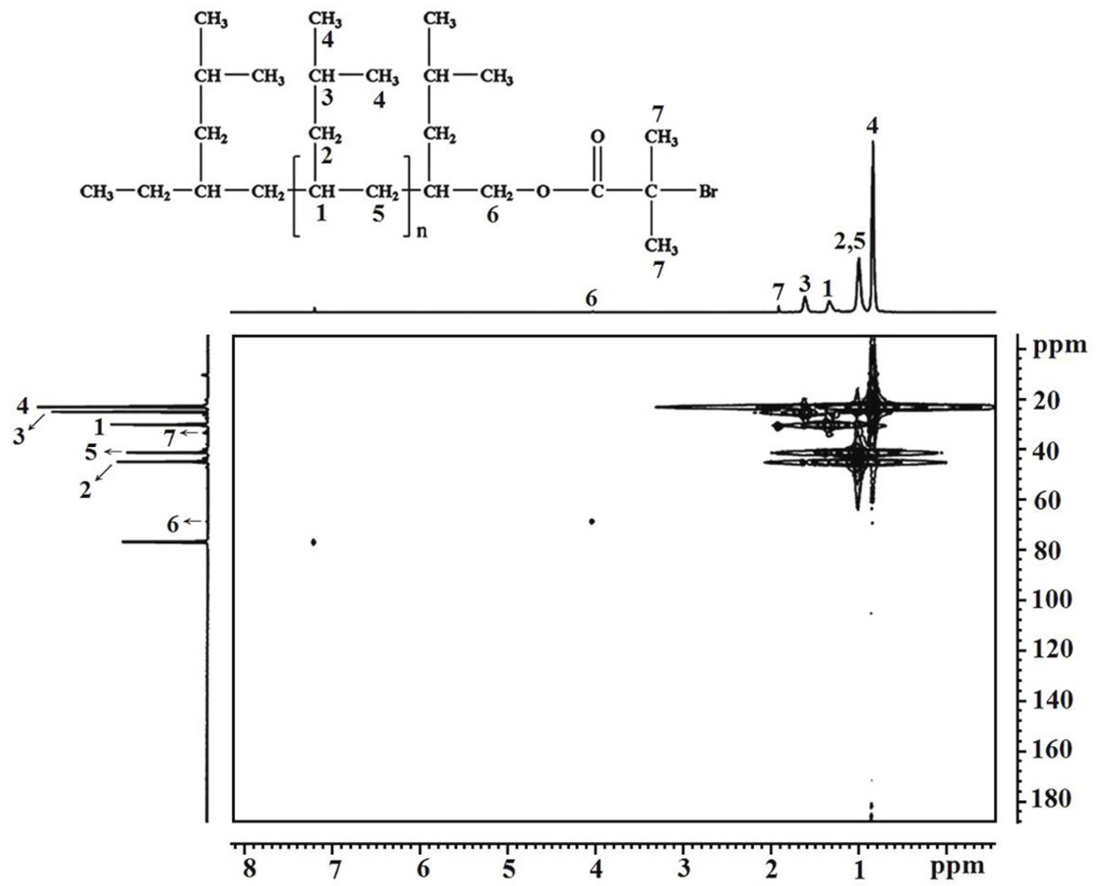

Figure S4. ^{1}H-^{13}C HMQC spectrum of sP4M1P-bromoester (M_n = 10500 g/mol, M_w/M_n = 1.39) (solvent: $CDCl_3$; temperature: 60°C).

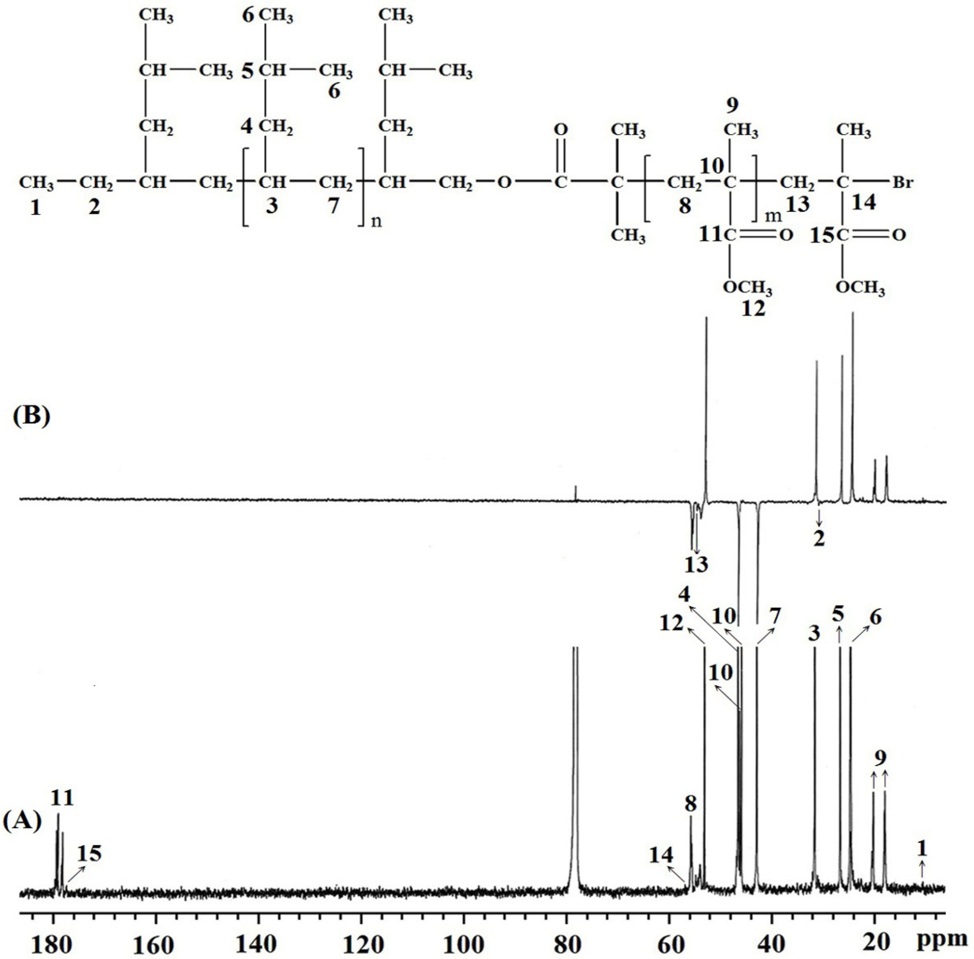

Figure S5. ^{13}C and ^{13}C (DEPT135) NMR spectra (125 MHz) of sP4M1P-*b*-PMMA (solvent: $CDCl_3$; temperature: 60°C).

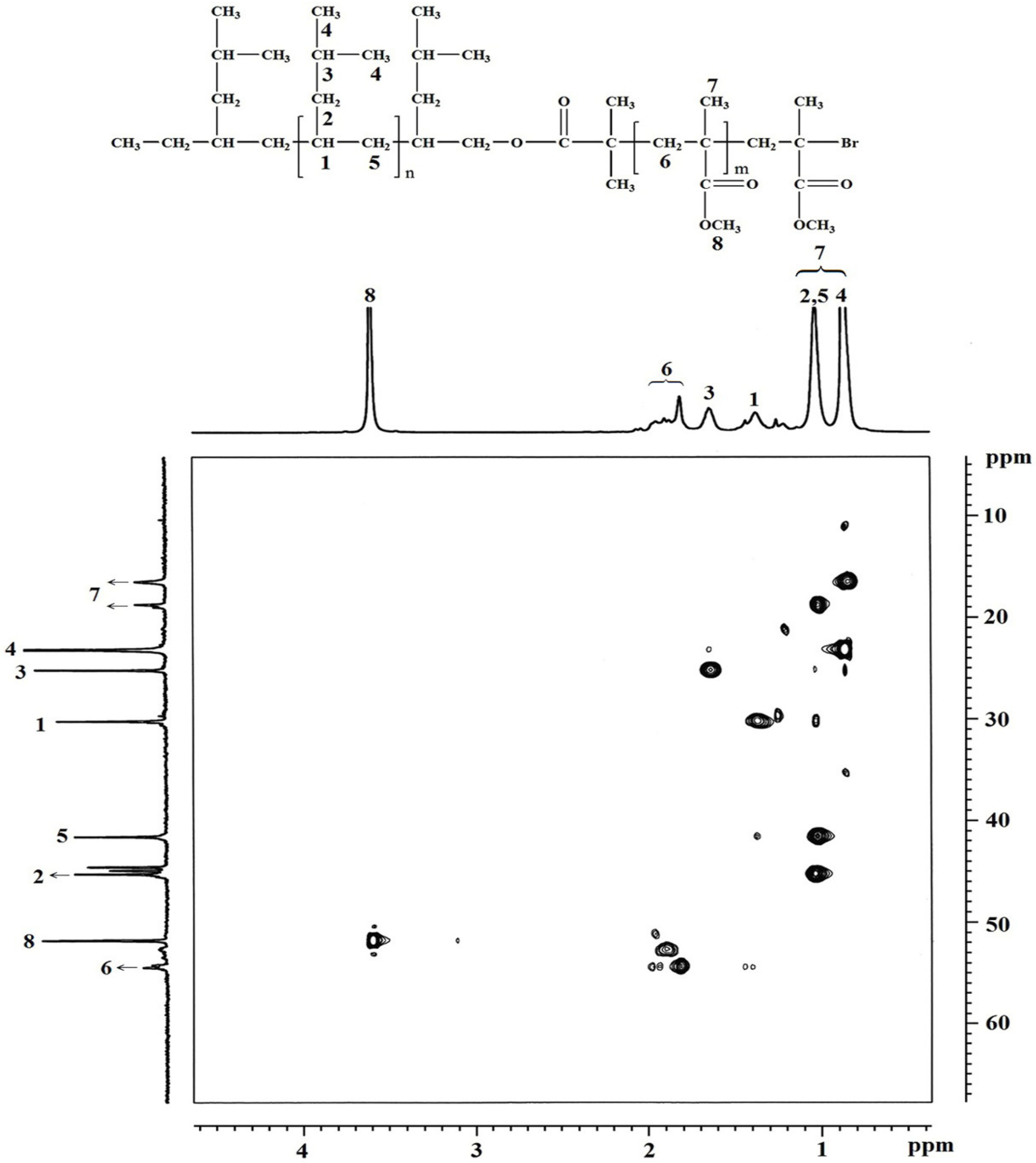

Figure S6. ^{1}H-^{13}C HMQC spectra of sP4M1P-*b*-PMMA (solvent: $CDCl_3$; temperature: 60°C).

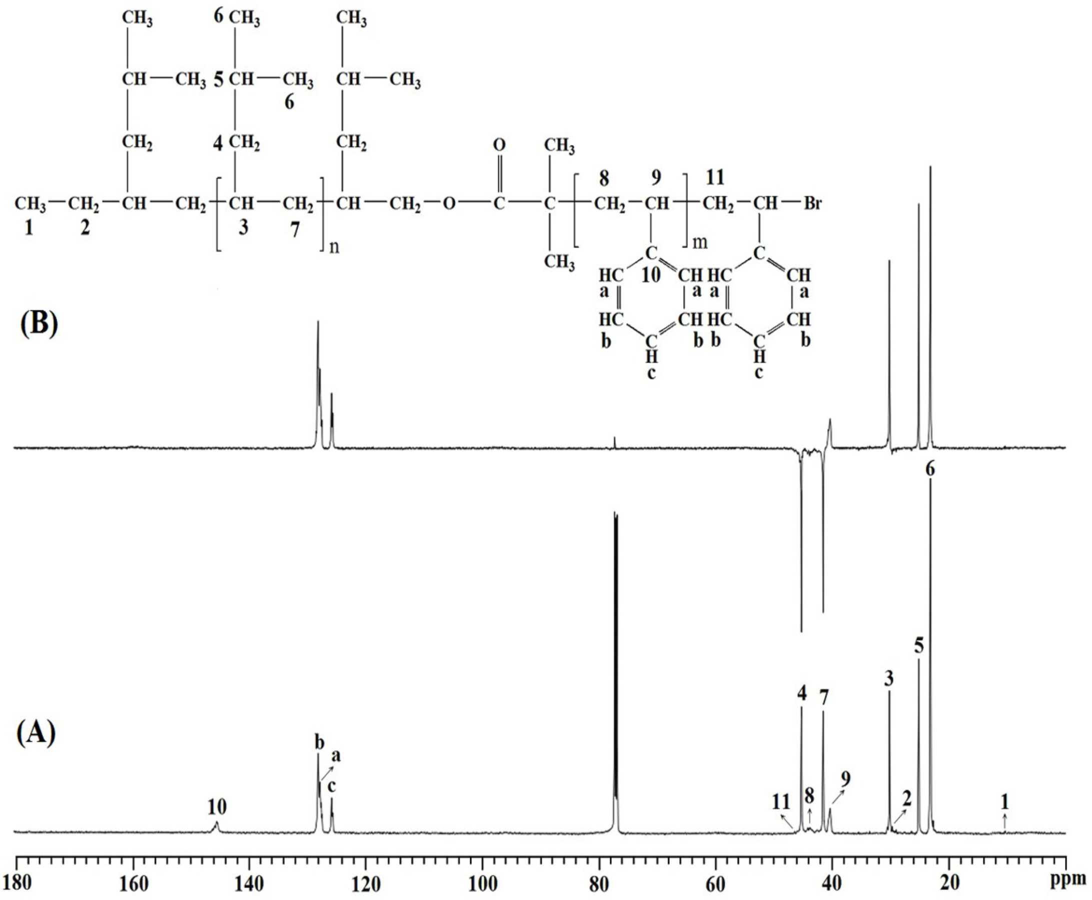

Figure S7. ^{13}C and ^{13}C (DEPT135) NMR spectra (125 MHz) of sP4M1P-*b*-aPS (solvent: $CDCl_3$; temperature: 60°C).

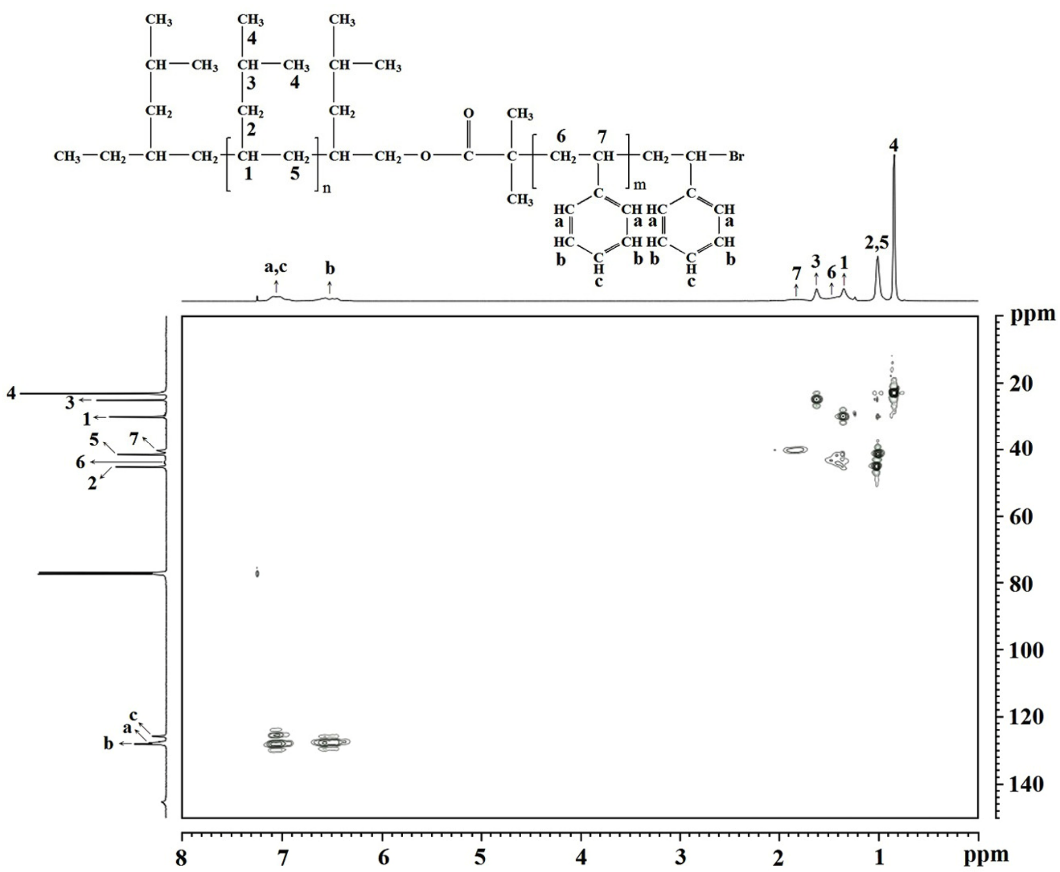

Figure S8. ^{1}H-^{13}C HMQC spectrum of sP4M1P-*b*-aPS (solvent: $CDCl_3$; temperature: 60°C).

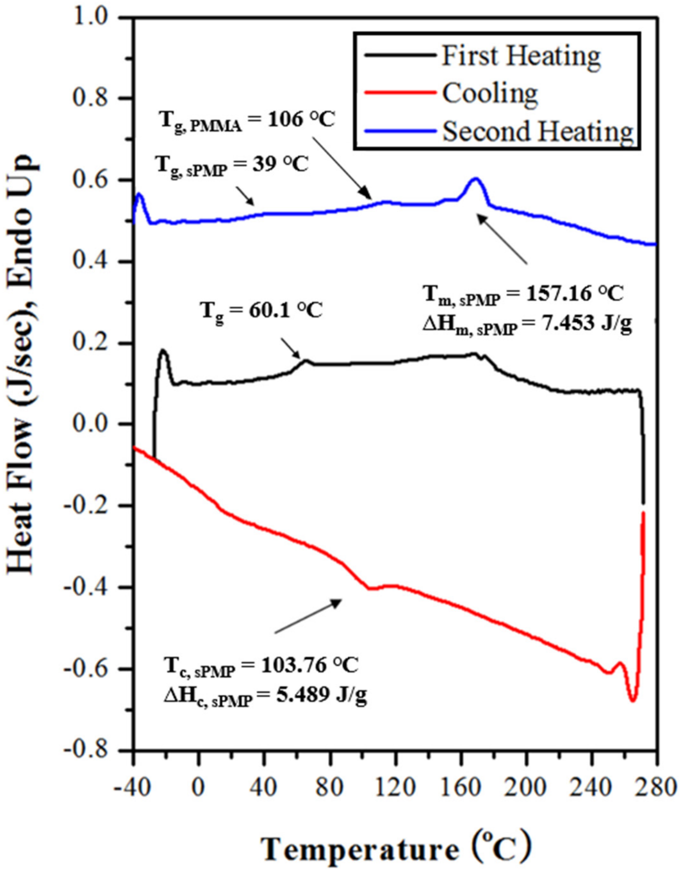

Figure S9. DSC heating thermograms of sP4M1P-*b*-PMMA.

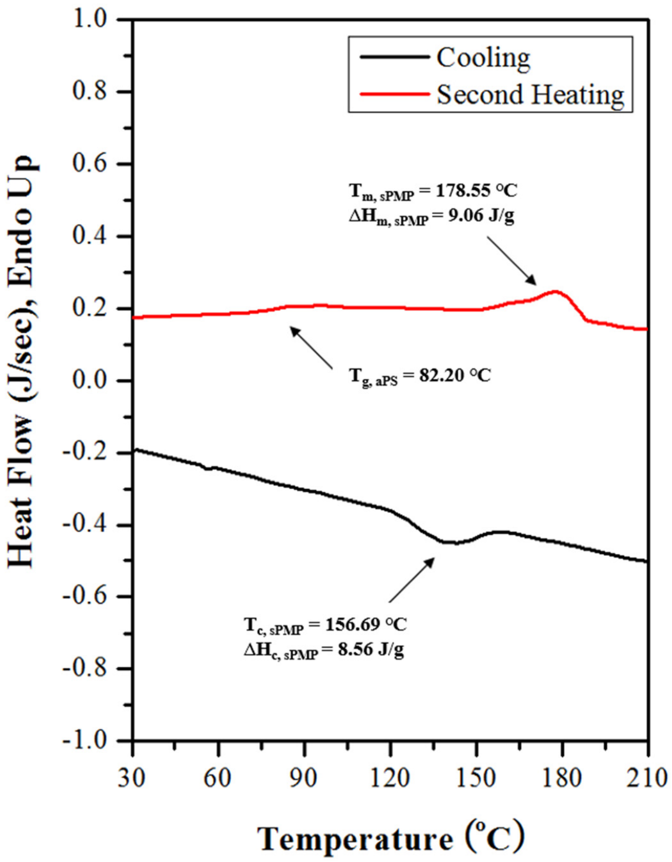

Figure S10. DSC heating thermograms of sP4M1P-*b*-aPS.
